# A consensus view of the proteome of the last universal common ancestor

**DOI:** 10.1002/ece3.8930

**Published:** 2022-06-03

**Authors:** Andrew J. Crapitto, Amy Campbell, AJ Harris, Aaron D. Goldman

**Affiliations:** ^1^ 6167 Department of Biology Oberlin College Oberlin Ohio USA; ^2^ 6572 Perelman School of Medicine University of Pennsylvania Philadelphia Pennsylvania USA; ^3^ Key Laboratory of Plant Resources Conservation and Sustainable Utilization South China Botanical Garden Chinese Academy of Sciences Guangzhou China; ^4^ 6167 Blue Marble Space Institute of Science Seattle Washington USA

**Keywords:** ancient genomes, ancient life, ancient metabolism, early evolution, last universal common ancestor, LUCA

## Abstract

The availability of genomic and proteomic data from across the tree of life has made it possible to infer features of the genome and proteome of the last universal common ancestor (LUCA). A number of studies have done so, all using a unique set of methods and bioinformatics databases. Here, we compare predictions across eight such studies and measure both their agreement with one another and with the consensus predictions among them. We find that some LUCA genome studies show a strong agreement with the consensus predictions of the others, but that no individual study shares a high or even moderate degree of similarity with any other individual study. From these observations, we conclude that the consensus among studies provides a more accurate depiction of the core proteome of the LUCA and its functional repertoire. The set of consensus LUCA protein family predictions between all of these studies portrays a LUCA genome that, at minimum, encoded functions related to protein synthesis, amino acid metabolism, nucleotide metabolism, and the use of common, nucleotide‐derived organic cofactors.

## Graphical abstract

Over the past couple of decades, researchers have used genomic data from organisms across the tree of life to infer the characteristics of the last universal common ancestor, an organism or community of organisms that lived roughly 3.5–4 billion years ago. Here, we compare predictions between previously published studies of the genome and proteome of the last universal common ancestor, measuring their agreement and identifying consensus between them.

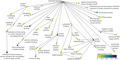

## 1 INTRODUCTION

The last universal common ancestor of life, or LUCA, represents an organism or population of organisms that is inferred to have existed prior to 3.5 billion years ago (Betts et al., 2018; David et al., 2018; Wolfe & Fournier, 2018). Even before the genomic era, a universal ancestry of all organisms was apparent through the discovery that all life shares a common biochemistry composed of nucleic acids, proteins, carbohydrates, lipids, and a small handful of other compounds (Kluyver & Donker, 1926; Weiss & Thauer, 1993). The LUCA as a common ancestor to all life was further supported by the discovery of a common canonical genetic code among all known organisms (Crick, 1968; Woese, 1965), with only small variations present in a handful of lineages (Knight et al., 2001). The observation that certain gene families are present in the genomes of every organism made it possible to demonstrate this common ancestry (Woese & Fox, 1977) through phylogenetic trees (Ciccarelli et al., 2006; Hug et al., 2016; Pace, 1997; Theobald, 2010). Paralogous gene families resulting from pre‐LUCA gene duplications allowed for a rooting of the universal tree between the bacterial clade and the archaeal/eukaryotic clade (Gogarten & Taiz, 1992; Gribaldo & Cammarano, 1998).

The unity of biochemistry, the shared genetic code, and the molecular functions of known universal genes, depict a LUCA that looked much more like a modern organism than the kind of simple replicator usually imagined in origin of life scenarios. The universality of the genetic code suggests that the LUCA had a recognizable translation system that encoded the 20 proteinogenic amino acids in codons composed of RNA. The unity of biochemistry, too, suggests that the LUCA had at least a somewhat elaborate set of metabolic and physiological processes. Some universal genes include components of the ATP synthase motor, the signal recognition particle system, and the Sec translocation channel, which together suggests that the LUCA had a fluid mosaic membrane composed primarily of lipids, but that also contained membrane‐bound proteins (Gogarten & Taiz, 1992; Gribaldo & Cammarano, 1998; Harris & Goldman, 2021).

Given that the LUCA appears to have been a relatively complex cellular organism rather than a simple replicator, a significant amount of evolutionary change must have occurred between the origin of life and the time of the LUCA. Two explanations that are not mutually exclusive could account for a relatively complex LUCA. One explanation is that random horizontal gene transfer was so rampant in the very earliest stages of evolutionary history, that it caused any diverging lineages to merge, thereby disrupting vertical inheritance. Under this view, the LUCA represents an evolutionary stage in which vertical inheritance was strong enough that divergence between the ancestors of bacteria and archaea could have taken place (Woese, 2002). A second explanation is that the organisms representing the LUCA lived alongside many other organisms, but over the subsequent 3.5–4 billion years of evolution, the descendent lineages of the other populations went extinct (Zhaxybayeva & Gogarten, 2004). Recent evidence suggests that both of these explanations are correct; the LUCA did indeed result from horizontal gene transfer between heterogeneous lineages, but the LUCA lineage was also the sole survivor of multiple contemporaneous lineages (Fournier & Alm, 2015).

The availability of genomic sequences from organisms across the tree of life has made it possible to infer detailed characteristics of the LUCA’s genome (Becerra et al., 2007). To do so, these studies first survey genomes or proteomes from a representative set of organisms across the tree of life and then apply evolutionary models or assumptions in order to infer whether the distribution of certain genomic or proteomic features in extant organisms is best explained by inheritance from the genome of the LUCA. These studies vary in what type of genomic or proteomic features are examined for ancestry in the LUCA. Several studies have focused on gene families (Harris et al., 2003; Mirkin et al., 2003; Weiss et al., 2016), while others examined protein domains and motifs (Delaye et al., 2005), protein structural architectures (Ranea et al., 2006; Wang et al., 2007), or the molecular functions of proteins (Srinvasan & Morowitz, 2009).

Inferring features of the LUCA genome or proteome is inherently challenging given the billions of years that have passed since the organism or community of organisms representing the LUCA existed on Earth. During that time, gene families may have been lost in one or another lineage. If this gene loss was extensive enough, it could have erased evidence that the gene family was present in the LUCA. In other words, such gene loss would mean that descent from the LUCA would not be the most parsimonious inference from the phylogeny of the gene family. In some cases, a gene family may be replaced by another unrelated gene family encoding proteins that perform a similar function (Koonin et al., 1996). Proteins related to the cell membrane and the DNA genome provide two likely examples of this phenomenon. Phospholipid bilayer membranes and DNA‐based genomes are both universal features of all extant life. However, many of the proteins involved in synthesizing and maintaining these foundational components of organisms are not universally distributed across the tree of life, likely due to gene loss and displacement that occurred soon after the divergence of the LUCA into the ancestors of archaea and bacteria (Goldman & Landweber, 2012; Peretó et al., 2004). Despite convergence on common functions, like, for example, the synthesis of deoxyribonucleotides (Reichard, 1993) or the replication of DNA (Forterre, 2002), the lack of a single universal gene family encoding those functions makes it difficult to ascribe them to the genome of the LUCA.

Given these limitations, studies that use extant genomes and proteomes to infer characteristics of the LUCA are, at best, inferring a minimal genome or proteome that cannot include those gene and protein families that may have been present in the LUCA but lost during subsequent evolution. In addition, each of the studies listed above is also constrained by the data available at the time that it was performed. Furthermore, without a broadly agreed upon set of best practices, the methodological approaches adopted by each study is unique from the others. However, even if individual studies have inherent methodological biases or otherwise produce some erroneous predictions, they should agree with one another if they are indeed accurately inferring the genome or proteome of the LUCA. As such, consensus among them should provide a more accurate portrayal of the LUCA genome (Goldman et al., 2012).

Consensus predictions have recently been used in several studies as a proxy for the minimal LUCA proteome. For example, Goldford et al. (2017) used the consensus predictions of LUCA genome or proteome studies as evidence that a metabolic network established from prebiotically available compounds was evolutionarily related to the early metabolism of the LUCA. Blanco et al. (2018) investigated nonbiological protein‐nucleic acid complexes to understand which amino acids may have played an important role during an RNA world, comparing their resulting amino acids to those of proteins from a consensus of LUCA genome and proteome studies. While these consensus predictions have been a useful proxy for the LUCA genome used by these and other studies (Goldman et al., 2012, 2016), this consensus, itself, has yet to be evaluated on its own. Here, we investigate the agreement between eight previously published studies of the LUCA genome or proteome and, based on our results, apply the consensus to infer several broad properties of the LUCA.

## 2 METHODS

### 2.1 Last universal common ancestor genome prediction mapping

The results of eight previously published LUCA genome studies were mapped onto the clusters of proteins from the eggNOG database. The results of six of these studies were previously mapped onto UniProt accessions as part of the original LUCApedia database (Delaye et al., 2005; Goldman et al., 2013; Harris et al., 2003; Mirkin et al., 2003; Srinivasan & Morowitz, 2009; Wang et al., 2007; Yang et al., 2005). The results of two additional studies were subsequently mapped onto UniProt accessions: Ranea et al. (2006) and Weiss et al. (2016). Results from the Ranea et al. study were mapped onto UniProt accessions using seed Protein Data Bank IDs corresponding to predicted LUCA CATH superfamilies, which were made available through supplementary data files published along with the study. Results from the Weiss et al. study were similarly mapped onto UniProt accessions using the COG IDs corresponding to predicted LUCA protein families made available through supplementary data files published along with the study.

UniProt accessions corresponding to all eight studies were mapped onto eggNOG clusters through the mapping file <uniprot‐15‐May‐2015.LUCA.tsv>, downloaded from the eggNOG FTP site. Only eggNOG clusters with at least one manually annotated accession, that is, from the Swiss‐Prot database (Boutet et al., 2016), were included in the subsequent analyses. Additionally, only eggNOG clusters categorized as OGs_LUCA were considered in the analyses because these clusters contain proteins from more than one domain of life.

### 2.2 Determining agreement between last universal common ancestor genome study predictions

Agreement between the predictions of LUCA genome studies was assessed using both inter‐rater tests and pairwise comparisons. Calculation of expected disagreement for the Krippendorff's α statistic was determined by replacing the actual eggNOG clusters associated with the individual study under consideration with randomly selected eggNOG clusters, performing an inter‐rater test on this new dataset, repeating this process 100 times, and taking the average over these iterations. For each pairwise comparison, Jaccard's similarity index was calculated by dividing the number of shared eggNOG clusters associated with both studies by the total number of eggNOG clusters associated with either study.

### 2.3 Functional analysis of consensus predictions of the last universal common ancestor proteome

Consensus predictions of the LUCA proteome were defined as any eggNOG cluster that four or more individual studies predicted to be present in the LUCA proteome. Gene Ontology (GO) terms associated with each eggNOG cluster were identified as any GO term linked to any UniProt accession in the SwissProt database that was a member of the particular eggNOG cluster. GO term assignments for the consensus predictions of the LUCA genome were performed by first adding the parent terms of GO terms associated with the consensus predictions of the LUCA genome to the list via the go.obo database available from the Gene Ontology webserver and then making the list nonredundant for each eggNOG cluster.

In order to determine which GO terms were significantly enriched in the consensus LUCA genome, random clusters were picked from the Swiss‐Prot database using the *random* python library, and the same process of gathering GO terms was then run on these clusters. This random selection was repeated 10^6^ times and the average and standard deviation was calculated for the expected frequency of each GO term. This average expected frequency and the associated standard deviation for each GO term was used to calculate a *p*‐value for each of the actual consensus LUCA GO terms. Averages, standard deviations, and *p*‐values were calculated using the *statistics* python library.

Ancestral enzyme functions were inferred from the consensus LUCA eggNOG clusters by first identifying enzyme commission numbers associated with specific proteins within each cluster as described in Gagler et al. (2022). Only annotations from reviewed UniProt accessions within the eggNOG clusters were included in this analysis. Out of the 366 consensus LUCA eggNOG clusters, 310 included at least one reviewed UniProt accession with an Enzyme Commission (EC) codes annotation. In order to roughly determine which enzyme functions were ancestral in each eggNOG cluster, as opposed to a more recent neofunctionalization, NCBI Taxonomic IDs were identified for each reviewed UniProt accession with an associated EC code. If an eggNOG cluster contained only one enzyme function, that enzyme function was only included if it was found in proteins spanning at least two taxonomic domains (from among Bacteria, Archaea, and Eukarya). If a single eggNOG cluster contained more than one enzyme function, only enzyme functions with the broadest taxonomic domain representation were included. This analysis resulted in 200 EC codes from 199 eggNOG clusters. The list of ancestral enzyme functions was then made nonredundant and all incomplete EC codes (i.e., those containing at least one dash in place of a digit) were removed. This further culling resulted in a list of 169 nonredundant EC codes. We then performed metabolic network and pathway analyses using the mapping tool on the KEGG database web server (Kanehisa & Sato, 2020; Kanehisa et al., 2021).

## 3 RESULTS

### 3.1 Mapping last universal common ancestor study predictions onto protein database accessions

We mapped genome or proteome predictions for the LUCA from eight different studies onto a common database framework in order to compare them directly to one another (Appendix S1). Specifically, we first mapped individual proteins to Uniprot accessions (The UniProt Consortium, 2019, 2021), which we subsequently aggregated into clusters of homologous proteins in the eggNOG database (Huerta‐Cepas et al., 2019). We consider these clusters of proteins in the eggNOG database to be an approximation of biological protein families. The protein cluster mapping provided by the eggNOG database is more informative for our purposes than the individual protein mapping to Uniprot accessions. If one study predicts a protein to have been present in the LUCA and another study predicts a different, but related, protein to have been present in the LUCA, both studies would actually be predicting that the same protein family was present in the LUCA, but their predictions would not match at the level of individual proteins. For example, a study that uses the SCOP database (Andreeva et al., 2007; Murzin et al., 1995) as its source database will have a different set of individual proteins available to it than one that uses the COG database (Tatusov et al., 2000) as its source database. Therefore, the specific proteins predicted by each study may not match one another, but they may belong to the same protein family and thereby belong to the same eggNOG cluster.

General information about the eight studies and their predictions for the genome or proteome of the LUCA is shown in Table 1. One noteworthy feature of this table is the large variation in the number of eggNOG clusters associated with the predictions for each study, ranging from 110 to 2078 clusters. Part of this variation could be due to the fact that some studies focus on broadly defined components of proteins or genes (like domains, motifs, or structural folds) rather than families of complete genes or proteins. In some cases, this will lead to a greater number of predictions when these components of proteins are mapped onto databases of complete proteins or protein families (in this case Uniprot and eggNOG). Specifically, Delaye et al. (2005) examined protein domains and motifs stored in the Pfam database (El‐Gebali et al., 2019; Punta et al., 2012). Yang et al. (2005) and Wang et al. (2007) examined protein structural folds and superfamilies stored in the SCOP database (Andreeva et al., 2007; Murzin et al., 1995; Ranea et al., 2006) examined protein structural topologies stored in the CATH database (Orengo et al., 2002; Sillitoe et al., 2015).

**TABLE 1:** Individual LUCA genome studies and their correspondence with eggNOG clusters of homologous proteins

LUCA genome study	Number of predictions and source database	Taxonomic genera surveyed^a^	Number of eggNOG clusters corresponding to LUCA predictions	Number of eggNOG clusters corresponding to the consensus LUCA predictions^b^
Harris et al. (2003)	80 COGs	31	110	81
Mirkin et al. (2003)	571 COGs^c^	26	848	304
Delaye et al. (2005)	114 Pfam domains	20	1259	302
Yang et al. (2005)	66 SCOP folds superfamilies	122	609	230
Ranea et al. (2006)	140 protein structures representing CATH superfamilies	71	119	76
Wang et al. (2007)	165 SCOP folds	91–153^d^	2078	345
Srinivasan and Morowitz (2009)	206 Enzyme Commission codes (via KEGG)	4	794	209
Weiss et al. (2016)	336 COGs (via GenBank)^e^	612	328	117

^a^ Genera were determined based on reconciling species reportedly sampled in each paper with the NCBI Taxonomy Database (Federhen, 2012).

^b^ Consensus LUCA predictions refers to eggNOG clusters associated with the predictions of four or more LUCA genome studies.

^c^ The article offers a range of possible predictions. We used the dataset derived from the authors’ gain penalty of 1 (i.e., equal weights assigned to a gain and a loss), which is the focus of their own analysis.

^d^ This range is based on a discrepancy between the article reporting that 185 genomes were sampled for the study and the specific genomes that could be ascertained based on the figures in the article that provided information on specific taxonomic sampling made available by the authors. The figures showed 123 unique species, which we determined belonged to 91 unique genera based on taxonomic reconciliation. If the remaining, missing 62 genomes belonged entirely to unique genera, the total number of genera would be 153. In contrast, if the missing genomes represented no additional, unique genera then the number of unique genera would be 91.

^e^ Protein families within this study were determined by sequence searches and were not provided in the article's supporting information. However, COGs were associated with 336 out of the 355 protein families predicted by the authors to have been present in the LUCA.

Many proteins contain multiple motifs, domains, and structural folds, and these features of proteins evolve semi‐independently from the protein coding genes within which they reside (Chothia et al., 2003). As such, even a small set of predicted protein motifs, domains, and structural folds may be represented across a large number of eggNOG clusters. For example, the P‐loop containing NTP hydrolase domain predicted by Delaye et al. (2005), Yang et al. (2005), Ranea et al. (2006), and Wang et al. (2007) to have been present in the LUCA, is represented in 325 different eggNOG clusters that contain at least one member from the reviewed Swiss‐Prot database (O’Donovan et al., 2002).

The results presented by Ranea et al. (2006) include both the individual CATH superfamilies and the corresponding seed protein structures (as Protein Data Bank IDs) that were used to search across proteomes for those superfamilies. To avoid the kind of over‐prediction described above, we perform subsequent analysis using only the seed protein structures from the Ranea et al. prediction set. Since three other studies focus on protein domains or motifs rather than whole proteins, if we did include any protein with a predicted CATH superfamily from the Ranea et al. study, consensus between the eight studies could be reached simply through a shared protein domain rather than membership in an ostensibly ancient protein family.

### 3.2 Agreement between last universal common ancestor proteome predictions

After mapping predictions from the eight previously published LUCA genome or proteome studies described above, we sought to identify the level of overall agreement and the areas of consensus among them. In order to gauge the level of agreement between studies, we used a series of inter‐rater tests (Cohen, 1960; Posner et al., 1990). Here, each inter‐rater test measured the level of agreement between the predictions of an individual study and the consensus among the other seven studies. This overall agreement between a single study and the consensus of the remaining studies can be measured directly as a percentage of matching predictions, or by more specialized statistical measures, such as Krippendorff's α (Equation 1) (Krippendorff, 1970) and Scott's π (Equation 2) (Scott, 1955). In this case, Krippendorf's α and Scott's π are mathematically equivalent and are both normalized to an expected value so that a score of one represents perfect agreement and a score of zero indicates a random level of agreement.

(1) $$\alpha =1‐\frac{D_{o}}{D_{e}},$$

where *D_o_* is the observed disagreement between studies and *D_e_* is the expected disagreement between studies.

(2) $$\pi =\frac{\mathrm{Pr}(a)‐\mathrm{Pr}(e)}{1‐\text{Pr}(e)},$$

where Pr(*a*) is the observed frequency of agreement between studies and Pr(*e*) is the expected frequency of agreement between studies.

The results of multiple inter‐rater tests are shown in Table 2. Each individual study was subjected to eight total inter‐rater test variants, where the score was calculated using either percent agreement or Krippendorff's α/Scott's π and where consensus was defined as agreement between two, three, four, or five of the remaining seven studies. Only positive predictions, *that is*, that a gene family was present in the genome of the LUCA, were considered. This was necessary because the majority of eggNOG clusters were not predicted by any of the eight studies to have been present in the LUCA. Including these negative predictions in the inter‐rater tests would, therefore, have led to uninformatively high scores for every study.

**TABLE 2:** Inter‐rater test scores for the predictions of individual LUCA genome studies (referred to by the last name of the primary author)

Statistic	Consensus threshold	Harris	Mirkin	Delaye	Yang	Ranea	Wang	Srinivasan	Weiss
Percent agree‐ment	2	0.86	0.57	0.39	0.61	0.81	0.30	0.46	0.54
	3	0.73	0.36	0.24	0.38	0.63	0.17	0.26	0.35
	4	0.56	0.16	0.10	0.17	0.45	0.06	0.10	0.19
	5	0.29	0.05	0.03	0.06	0.21	0.02	0.02	0.07
Krippen‐dorff’s α / Scott’s π	2	0.68	0.15	‒0.08	0.19	0.55	‒0.05	‒0.09	‒0.03
	3	0.65	0.24	0.13	0.24	0.52	0.09	0.12	0.18
	4	0.54	0.14	0.09	0.15	0.41	0.05	0.08	0.15
	5	0.29	0.05	0.03	0.06	0.20	0.02	0.02	0.07

Note

Heatmap index: 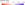

Taken together, the results of these inter‐rater tests show strong agreement for some studies and weak agreement for others (sometimes equivalent to random chance). The inter‐rater scores differ from test to test, but the rank of individual studies is very stable, with Harris et al. (2003) and Ranea et al. (2006) having the highest and second highest inter‐rater scores, respectively, across all versions of the test. The highest scoring studies also have the least number of eggNOG clusters associated with their genome or proteome predictions and the overall negative correlation between prediction set size (Table 1) and inter‐rater score (Table 2) is strong. For example, the correlation (Pearson's *r*) between the number of eggNOG clusters associated with a study and the study's *α*/*π* score using a consensus level of four is −0.72.

We further investigated this effect of prediction set size on inter‐rater score by performing inter‐rater tests comparing both positive and negative predictions across all eight studies so that the total number of predictions made by each study would be identical (Appendix [Link], [Link]). While the actual scores of these inter‐rater tests are uninformatively high due to the majority of predictions across all studies being negative predictions, the rank order of scores across all forms of inter‐rater tests is mostly identical to the rank order of the inter‐rater tests using only positive predictions (average Spearman's correlation = 0.93, median Spearman's correlation = 0.94). We also performed similar inter‐rater tests excluding the smallest two prediction sets, Harris et al. (2003) and Ranea et al. (2006) (Appendix S6). Here, the inter‐rater scores for individual studies are worse than scores from the original set of inter‐rater tests, with no study yielding an *α*/*π* score above 0.02. These results suggest that studies with smaller prediction sets are not yielding higher inter‐rater scores because they are smaller *per se*, but because the predictions are more conservative and thus shared to a greater degree with the other studies.

In addition to the inter‐rater test, we also assessed the similarity between every pair of studies calculated as the Jaccard's similarity index between the eggNOG clusters associated with their LUCA genome or proteome predictions (Table 3). Jaccard's similarity is defined as the intersection (in this case, the number of matching eggNOG clusters between the two studies) divided by the union (in this case, the total number of unique eggNOG clusters predicted by both studies). Surprisingly, no study shows any notable level of similarity with any other. These pairwise similarity comparisons demonstrate that there is very little overall agreement between individual LUCA genome or proteome studies.

**TABLE 3:** Jaccard's similarity index between pairs of individual LUCA genome studies (referred to by the last name of the primary author)

	Harris	Mirkin	Delaye	Yang	Ranea	Wang	Srinivasan	Weiss
Harris	1	0.13	0.05	0.08	0.24	0.03	0.02	0.08
Mirkin		1	0.22	0.17	0.09	0.19	0.20	0.12
Delaye			1	0.15	0.06	0.19	0.17	0.09
Yang				1	0.06	0.27	0.11	0.09
Ranea					1	0.04	0.03	0.05
Wang						1	0.16	0.08
Srinivasan							1	0.08
Weiss								1

Note

Heatmap index: 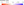

### 3.3 Consensus predictions of the last universal common ancestor proteome

The moderate to good performance of several studies on the inter‐rater tests compared to the broad lack of similarity between any pair of studies suggests that consensus among LUCA proteome studies may lead to a more accurate representation of the LUCA proteome than any individual study on its own. As such, we sought to generate a consensus of eggNOG clusters that are predicted by four or more studies to represent LUCA protein families and characterize the functional repertoire of the LUCA proteome based on these consensus predictions. We identified 366 eggNOG clusters that were predicted by four or more studies to have been present in the genome of the LUCA (Appendix S2). Not surprisingly, the studies with the largest prediction sets contributed the most predictions to this consensus set of eggNOG clusters (Table 1).

Notably, 3431 eggNOG clusters were predicted by at least one study to have been present in the genome of the LUCA while only one eggNOG cluster (COG0541) is predicted by all eight studies to have been present in the LUCA. This eggNOG cluster corresponds to the protein component of the signal recognition particle (Ffh in bacteria, SRP54 in archaea and eukaryotes). The Ffh/SRP54 protein is part of an ancient and highly conserved system responsible for the translocation of proteins across membranes in all three domains of life (Gribaldo & Cammarano, 1998; Harris & Goldman, 2021). This result is also in agreement with a study of universal, vertically inherited functional RNAs (Hoeppner et al., 2012), which finds that the SRP RNA is the only universal functional RNA not associated with the translation system.

### 3.4 Functional characterization of consensus last universal common ancestor proteome predictions

**FIGURE 1:**
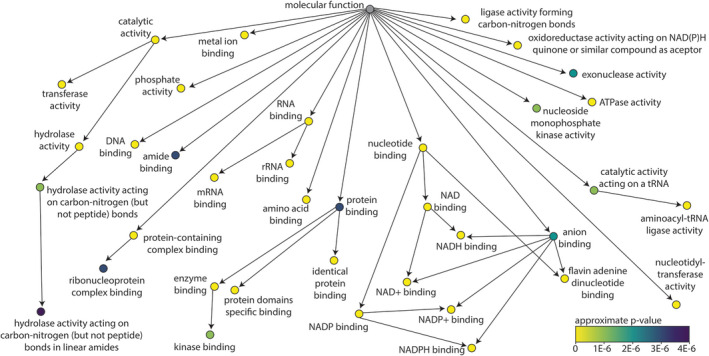
Statistically overrepresented Gene Ontology (GO) terms in the Molecular Function category associated with consensus LUCA eggNOG clusters. Arrows indicate parent‐child GO term relationships. All GO terms shown in the figure have an associated *p*‐value < 4 × 10^−6^, which is below the Bonferroni‐corrected threshold of *p* ≤ 4.6 × 10^−6^. Note that the term “Molecular Function,” itself, is not statistically significant. The terminal GO terms in each branch of the parent‐child network (i.e., the most specific GO terms) have been removed for clarity, but all statistically significant GO terms are available as Appendix S3

We next identified Gene Ontology (GO) terms (Ashburner et al., 2000; The Gene Ontology Consortium, 2019) associated with the consensus LUCA eggNOG clusters (Figure 1). The statistical significance of each GO term associated with the consensus LUCA eggNOG clusters was determined using permutation tests of all eggNOG clusters chosen at random. This resulted in 111 Molecular Function GO terms with Bonferroni‐corrected *p*‐values ≤ 4.6 × 10^−6^ (Figure 1, Appendix S3). Taken together, GO terms associated with the consensus LUCA eggNOG clusters most often relate to translation (e.g., mRNA binding, rRNA binding, and aminoacyl tRNA ligase activity), nucleic acids (e.g., DNA binding and exonuclease activity), and the use of nucleotide derived cofactors (e.g., NAD binding, flavin adenine dinucleotide binding, ATPase activity, and nucleoside monophosphate kinase activity).

**FIGURE 2:**
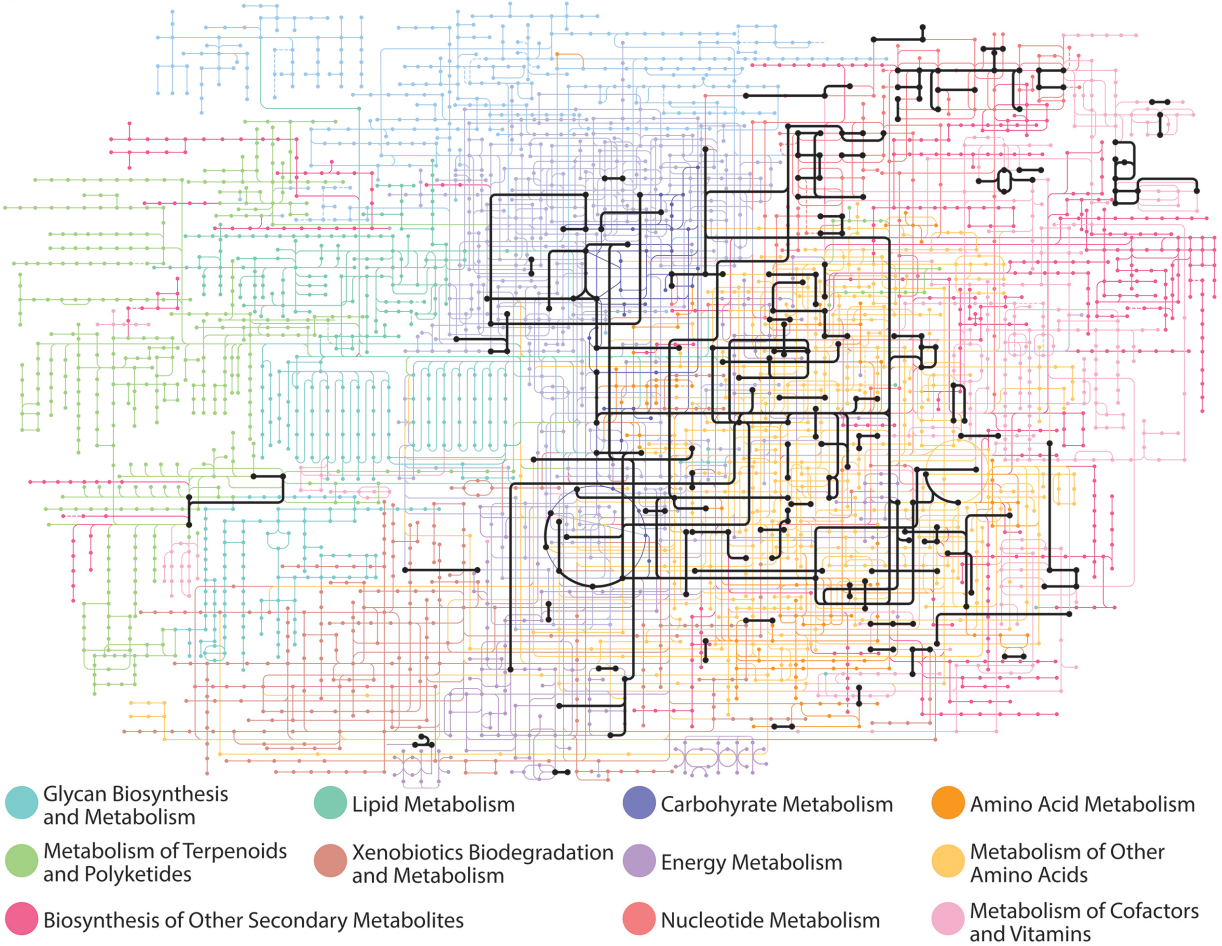
Ancestral enzyme functions determined from consensus LUCA eggNOG clusters mapped onto a universal metabolic network. The consensus LUCA enzyme functions are represented by 169 Enzyme Commission codes. The universal metabolic network and color‐coding of metabolic categories are from the global Metabolic Pathways network (map 01100) from the KEGG database (Kanehisa et al., 2017; Ogata et al., 1999). “Metabolism of Other Amino Acids” is terminology that the KEGG database uses to indicate amino acids that are not included in proteins, such as D‐amino acids

We also sought to characterize a core LUCA metabolism based on enzyme functions associated with the consensus LUCA eggNOG clusters. Consensus ancestral enzyme functions were defined as any Enzyme Commission (EC) code that was associated with proteins in a consensus LUCA eggNOG cluster wherein the same EC code was found in representative proteins across multiple taxonomic domains (Appendix S4). The resulting 169 unique enzyme functions (4‐term EC codes) were mapped onto the global metabolic pathways as defined by the KEGG database (Figure 2) (Kanehisa et al., 2017; Ogata et al., 1999). Mapping these enzyme functions onto the KEGG database allowed us to determine the functional overlap between modern metabolic pathways or networks and enzyme functions associated with the LUCA (which we refer to as “coverage,” below). From this perspective, the enzyme functions associated with the consensus LUCA proteome appear to converge on the broad metabolic categories of sugar, amino acid, and nucleotide metabolism with some coverage in the categories of energy and cofactor metabolism. Consensus ancestral enzyme functions show almost no coverage of lipid metabolism, starch metabolism, or the metabolism of other large biomolecules (e.g., terpenoids and polyketides).

At the level of individual pathways (Table 4), the pathway most closely associated with enzyme functions in the consensus LUCA proteome is the aminoacyl‐tRNA biosynthesis pathway (KEGG map00970), in which 58% of all pathway enzymes are associated with the consensus LUCA proteome. The other most prominent pathways pertain to amino acid metabolism and nucleotide metabolism with glycolysis/gluconeogenesis also showing a high degree of coverage. Curiously, the Drug Metabolism pathway (map00983) shows the third highest percent coverage by consensus LUCA enzyme functions, a result similar to one observed by Goldman et al. (2012). The enzyme functions of the consensus LUCA proteome found in the Drug Metabolism pathway are exclusively involved in nucleobase chemistry associated with two drugs that both contain nucleobases, 6‐thioguanosine monophosphate synthesis, and fluorouracil. These same enzyme functions are found in nucleotide metabolism pathways, and this is likely the cause of their inclusion in the set of consensus LUCA enzyme functions.

**TABLE 4:** Metabolic pathway coverage of enzyme functions associated with consensus LUCA eggNOG clusters

KEGG pathway	Number of EC code matches	Percentage of total pathway EC codes (%)
Aminoacyl‐tRNA biosynthesis (map00970)	18	58
Valine, leucine, and isoleucine biosynthesis (map00290)	5	36
Drug metabolism—other enzymes (map00983)	7	28
Alanine, aspartate, and glutamate metabolism (map00250)	13	26
Pyrimidine metabolism (map00240)	15	24
Lysine biosynthesis (map00300)	8	24
Carbon fixation in photosynthetic organisms (map00710)	6	24
Phenylalanine, tyrosine and tryptophan biosynthesis (map00400)	9	24
Arginine biosynthesis (map00220)	7	23
Glycolysis/Gluconeogenesis (map00010)	11	22
Purine metabolism (map00230)	18	17
Histidine metabolism (map00340)	6	15
Glycine, serine, and threonine metabolism (map00260)	10	14
Nitrogen metabolism (map00910)	5	13
Cysteine and methionine metabolism (map00270)	9	11
Carbon fixation pathways in prokaryotes (map00720)	5	10
Pentose phosphate pathway (map00030)	5	9
Methane metabolism (map00680)	8	9
Pyruvate metabolism (map00620)	6	8
Arginine and proline metabolism (map00330)	6	7
Amino sugar, and nucleotide sugar metabolism (map00520)	6	5

## 4 DISCUSSION

We undertook this study with the hypothesis that the specific predictions of the various studies on the LUCA genome or proteome performed over the last two decades would largely agree with one another. We expected that each study should have some unknowable level of error because it is inherently difficult to infer specific details about life forms that existed at least 3.5 billion years ago. However, if the previously published LUCA genome or proteome studies are at all accurate, they should agree with one another and, because these studies have used largely independent approaches to infer features of the LUCA genome and proteome, agreement between them could be taken as support for this sort of approach.

Surprisingly, we found that studies of the genome or proteome of the LUCA do not uniformly agree with one another. Inter‐rater tests show that the fewer predictions a study makes, the higher the inter‐rater scores. One possible explanation for this trend is that it is an artifact of only comparing positive predictions between studies. However, a similar inter‐rater test that compared both negative and positive predictions between studies (Appendix S5) yielded inter‐rater scores with similar rank orders of study scores to the original inter‐rater tests that compared only positive predictions. That is, the average Spearman's rank correlation (*ρ*) between the results of the original set of inter‐rater tests and these similar inter‐rater tests that include negative predictions was 0.93 (median = 0.94) with a range between 0.83–0.98 across all eight versions of the inter‐rater test.

The specific scores from this inter‐rater test that compared both negative and positive predictions are not as informative as those from the original inter‐rater tests that only compare positive predictions because a large majority of eggNOG clusters were not included in any of the LUCA datasets. But the similarity in the rank order of these scores with those of the original inter‐rater tests suggests that our observation that higher scores are associated with smaller studies is not simply an artifact of only comparing positive predictions. An alternative explanation, which we prefer, is that smaller prediction sets represent more conservative studies and thus have a higher likelihood of matching the consensus predictions of the other studies. This observation should not be taken to mean that more conservative studies are *de facto* better representations of the LUCA proteome. Despite having better agreement with the consensus of the other studies, these smaller studies contribute fewer predictions to that consensus (Table 1).

It is also informative that while some studies performed well on the inter‐rater tests, which compared their predictions to the consensus predictions from the other eight studies, none of them showed a high or even moderate level of similarity with any other individual study. Even the studies with fewest predictions, which we consider to be the most conservative, were not in strong agreement with each other. We take this as evidence that the consensus of studies may provide a more accurate account of the genome and proteome of the LUCA than any individual study.

To this end, we assembled a consensus set of eggNOG clusters that are each predicted by four or more studies to be associated with the core LUCA proteome. The core LUCA proteome derived from this consensus is composed of 366 eggNOG clusters. This is more than the number of eggNOG clusters associated with the two LUCA genome studies with the smallest prediction sets, but less than the average, 768, or the median, 702, number of eggNOG clusters across all eight LUCA genome studies. The molecular functions associated with the consensus minimal LUCA proteome generally revolve around sugar, amino acid, and nucleotide metabolism, the translation process, and the use of organic, nucleotide derived cofactors. We observe a lack of consensus LUCA enzyme functions associated with phospholipid metabolism, which would have been critical for cell membrane biosynthesis. However, we note that the only eggNOG cluster associated with all eight LUCA genome or proteome studies belongs to the Ffh/SRP54 family of proteins, which are involved in the process of protein translocation across membranes.

Several cell membrane‐related Gene Ontology terms from the categories of Cellular Component and Biological Function are also inferred from the consensus minimal LUCA proteome such as “Plasma Membrane” (GO:0005886) and “SRP‐dependent cotranslational protein targeting to membrane” (GO:0006614) (Appendix S3). However, the Gene Ontology categories of Biological Function and Cellular Component are less informative here than the Molecular Function category because potentially ancient proteins that performed functions relevant to ancient life will also be included in Biological Function and Cellular Component categories that are clearly not ancient. The categories of “SRP‐dependent Cotranslational Protein Targeting to Membrane” (GO:0006614) and “Plasma Membrane” (GO:0005886) share protein eggNOG clusters with “Mitochondrial Inner Membrane” (GO:0005743) and “Nuclear Membrane” (GO:0031965), which are clearly eukaryotic and therefore not relevant to the LUCA, but which are also statistically significant Gene Ontology terms in our analysis. As such, we report the Biological Function and Cellular Component Gene Ontology terms along with Molecular Function Gene Ontology terms in Appendix S3, but also suggest that the former two are not particularly informative.

The Molecular Function Gene Ontology terms associated with the LUCA largely confirm what previous analyses have proposed or determined about the LUCA and, more broadly, early life forms. For example, the translation process is well known to be ancient (Freeland et al., 1999; Goldman et al., 2010) and many of the proteins involved in translation machinery appear to predate the LUCA (Fournier & Alm, 2015; Fournier et al., 2012; Harris & Goldman, 2018). A corollary to the influential RNA world hypothesis (Gilbert, 1986; Visser, 1984) is that the translation system evolved within the context of an RNA‐based genetic system (Freeland et al., 1999). This idea has been supported by previous LUCA studies. Harris et al. (2003), for example, found that most universal COGs encode proteins that physically associate with the ribosome and those that do not are often involved with the translation process in some other way. Similarly, Hoeppner et al. (2012) found that nearly all universal, vertically inherited functional RNAs (save the SRP RNA) are involved in the translation system. Translation‐related genes or proteins are prevalent in the predictions of seven of the eight previously published LUCA genome or proteome studies analyzed here. The exception is the study of Srinivasan and Morowitz (2009) which compared metabolic enzymes across organisms and thereby may have failed to recover nonmetabolic processes in their predictions. Another corollary to the RNA world hypothesis is that nucleotide cofactors acted as a bridge between a ribozyme‐based metabolism and a protein enzyme‐based metabolism that used nucleotides or nucleotide derivatives to catalyze the same reactions (Goldman & Kacar, 2021; Huang et al., 2000; Kirschning, 2021; White, 1976). This potential residual of an RNA world is reflected in our finding that consensus LUCA eggNOG clusters show a significantly greater use of nucleotide‐derived cofactors.

Though our results are broadly consistent with previous work on the minimal LUCA genome or proteome, we believe this consensus view of the LUCA and its functional repertoire is more accurate than any one of the eight single studies that we analyzed. Our results do not preclude the possibility that one of the previously published LUCA genome or proteome studies is highly accurate, while the other seven are not. However, it is more likely that all of the previously published approaches have been constrained by the lack of availability of analytical resources, including abundant genomes from across the tree of life, to generate a robust evolutionary framework. This evolutionary framework, in the form of a taxonomically well‐sampled phylogenetic tree, facilitates studying individual protein families for their presence in the LUCA (Berkemer & McGlynn, 2020), by determining if their topologies match the species tree of life. This match between the gene tree and species tree, along with broad taxonomic sampling, should be considered both necessary and sufficient to accurately claim that the protein family was present in the LUCA. This more rigorous approach is sometimes, but not always, used when predicting the minimal genome or proteome of the LUCA. In some cases even when phylogenetic tree topologies were used to confirm the presence of a protein family in the LUCA, an insufficient number of genomes were available at the time to constitute a truly broad taxonomic sampling, *for example*, Harris et al. (2003) and Mirkin et al. (2003).

Despite these methodological constraints, reconstruction of the LUCA genome has been a useful exercise insofar as it has demonstrated that there is a conserved genomic core, mutually inherited by organisms across the tree of life. It was from these earliest studies that we first learned that the LUCA represented a complex stage of early evolution far beyond the simple replicators that followed the origin of life (Becerra et al., 2007). Future progress toward a more detailed understanding of the LUCA can be made by focusing on especially informative protein families or physiological processes that can be confidently attributed to the genome of the LUCA by methods that combine robust taxonomic sampling and careful analysis of gene tree topologies. Automating this more rigorous approach has yet to be accomplished, but has the potential to considerably advance our understanding of the minimal LUCA genome and proteome. Whether through advanced automated pipelines or careful and methodical work by a community of researchers, species tree‐aware phylogenetic analyses of shared protein families (e.g., via GeneRax (Morel et al., 2020) or other tools) are likely to yield an even more accurate and detailed characterization of the LUCA.

## AUTHOR CONTRIBUTIONS

**Andrew Crapitto:** Conceptualization (equal); Data curation (equal); Formal analysis (equal); Investigation (equal); Methodology (equal); Validation (equal); Writing – review & editing (equal). **Amy Campbell:** Conceptualization (equal); Methodology (equal); Visualization (supporting); Writing – review & editing (equal). **AJ Harris:** Conceptualization (equal); Data curation (equal); Investigation (equal); Methodology (equal); Writing – review & editing (equal). **Aaron Goldman:** Conceptualization (lead); Data curation (lead); Formal analysis (equal); Funding acquisition (lead); Investigation (equal); Methodology (equal); Project administration (lead); Supervision (lead); Validation (equal); Visualization (equal); Writing – original draft (lead); Writing – review & editing (equal).

## CONFLICT OF INTEREST

All authors declare that they have no conflicts of interest.

## Supporting information


Appendix S1

Appendix S2

Appendix S3

Appendix S4

Appendix S5

Appendix S6

## Data Availability

All data and intermediate results produced by this study are available as supplemental data files accessible via the journal. The same supplemental data files have also been archived on DRYAD at https://doi.org/10.5061/dryad.5hqbzkh7s.
