# Supplementary material for: A consensus view of the proteome of the last universal common ancestor
Source: Ecol Evol. 2022 Jun 3;12(6):e8930. doi: 10.1002/ece3.8930 (PMC9165204; doi:10.1002/ece3.8930)
Supplement: Supplementary file 5 — Appendix S5 [file ECE3-12-e8930-s001.pdf]

Scores from inter-rater tests similar to those shown in Table 2 except that both positive predictions and negative predictions were compared between each individual LUCA study and the consensus of the other seven LUCA studies.

Statistic	Consensus Threshold	Harris	Mirkin	Delaye	Yang	Ranea	Wang	Srinivasan	Weiss
Percent Agreement	%, 2	1.00	0.98	0.96	0.99	1.00	0.93	0.98	0.99
	%, 3	0.99	0.97	0.95	0.98	0.99	0.92	0.97	0.99
	%, 4	0.98	0.96	0.94	0.97	0.98	0.91	0.96	0.98
	%, 5	0.97	0.95	0.93	0.96	0.97	0.90	0.95	0.96
Krippendorff's α/ Scott's π	α/π, 2	0.97	0.51	0.03	0.68	0.95	-1.00	0.41	0.78
	α/π, 3	0.85	0.28	-0.20	0.45	0.83	-1.33	0.19	0.62
	α/π, 4	0.54	-0.01	-0.47	0.15	0.54	-1.62	-0.08	0.33
	α/π, 5	0.08	-0.30	-0.71	-0.16	0.11	-1.72	-0.36	-0.05

In this case, Krippendorf's α and Scott's π are mathematically equivalent…

$\alpha = 1 - \frac{Do}{De}$, where D_o is the observed disagreement between studies and D_e is the expected disagreement between studies.

$\pi = \frac{Pr\ (a) - Pr\ (e)}{1 - Pr\ (e)}$, where Pr(*a)* is the observed frequency of agreement between studies and Pr(*e)* is the expected frequency of agreement between studies.
