# Supplementary material for: A consensus view of the proteome of the last universal common ancestor
Source: Ecol Evol. 2022 Jun 3;12(6):e8930. doi: 10.1002/ece3.8930 (PMC9165204; doi:10.1002/ece3.8930)
Supplement: Supplementary file 6 — Appendix S6 [file ECE3-12-e8930-s002.pdf]

Scores from inter-rater tests similar to those shown in Table 2 except with the two smallest prediction sets, Harris *et al*. (2003) and Ranea *et al*. (2006) removed.

Statistic	Consensus Threshold	Mirkin	Delaye	Yang	Wang	Srinivasan	Weiss
Percent Agreement	%, 2	0.48	0.39	0.46	0.30	0.42	0.53
	%, 3	0.15	0.10	0.16	0.07	0.14	0.19
	%, 4	0.02	0.02	0.02	0.01	0.02	0.04
Krippendorff's α/ Scott's π	α/π, 2	0.02	-0.03	-0.08	-0.02	-0.10	-0.01
	α/π, 3	0.01	0.00	0.00	0.00	0.00	0.01
	α/π, 4	0.01	0.00	0.00	0.00	0.00	0.01

In this case, Krippendorf's α and Scott's π are mathematically equivalent…

$\alpha = 1 - \frac{Do}{De}$, where D_o is the observed disagreement between studies and D_e is the expected disagreement between studies.

$\pi = \frac{Pr\ (a) - Pr\ (e)}{1 - Pr\ (e)}$, where Pr(*a)* is the observed frequency of agreement between studies and Pr(*e)* is the expected frequency of agreement between studies.
